# Colic1Read before the February meeting of the Kansas State Veterinary Medical Association.

**Published:** 1897-08

**Authors:** E. P. Schaffter

**Affiliations:** Mount Eaton, Ohio


### COLIC.[1]

By E. P. Schaffter, V.S.,
MOUNT EATON, OHIO.

This term is applied generally to a variety of conditions giving rise to abdominal pain. In the horse there are no more common or more frequently fatal affections than the diseases known as spasmodic and flatulent colic, indigestion, constipation, volvulus, intussusception, and various other intestinal derangements. Among these I will briefly consider the first two, namely, spasmodic and flatulent colic.

Spasmodic colic is a spasmodic contraction of the muscular fibres of the intestines, not generally having a tendency to terminate in enteritis. It is usually due to some primary cause which interferes with the peristaltic movements of the bowels, and is sometimes brought on by a combination of circumstances. The most common causes are overfeeding and irregular feeding. In regard to overfeeding, as is usually the case with horses that are being prepared for Eastern market, they are crammed with feed, as they are generally cared for in a way that their appetite assumes a voracious nature. This condition is taken advantage of to prepare them for market speedily. Want of exercise and the above-mentioned cause not infrequently produce a feverish and arrested secretive condition of intestines, causing the bowels to become overloaded, hence the pain caused by nature's endeavor to remove this abnormality. Drinking excessively of cold water when the animal is warm is also quite a frequent cause of colic. I will not consider those

**The seat of the perfect reproduction of the ancient Parthenon, at Nashville.**

[1] Read before the February meeting of the Kansas State Veterinary Medical Association.

causes having their origin in derangements of the accessory organs of digestion, as the space in my paper will not permit of a detailed consideration of all the direct causes of this affection.

*Symptoms.* A careful study of the symptoms of this disease should be made in order to arrive at a correct diagnosis, as the symptoms are nearly identical in other conditions requiring different treatment.

Spasmodic colic always begins suddenly. If feeding, the animal stops suddenly, paws, shifts about, and crouches. He looks around at his flanks, sometimes attempts to bite himself; he will lie down and turn on his belly or roll on his back, and often, as the paroxysm is abating, he will lie outstretched on his side, as if to rest himself. He then rises, shakes himself, and appears well. He will then seek food. The subsidence of these symptoms is of but short duration, and they often reappear in an aggravated form. Sometimes the attacks diminish in violence, each subsequent attack being less until the animal recovers; but under other circumstances the pain becomes continuous, the pulse becomes quick and hard, the breathing accelerated, the eyes show an expression of anguish, becoming prominent and staring, and there is usually noticed a sympathetic derangement of the brain. When the disease has progressed to this stage it usually terminates fatally. There will be noticed a coldness of extremities, cold sweats bedewing the body, and twitching of the muscles of the body. In diagnosticating this disease you should always bear in mind the history of the disease, nature of the horse, the sudden violent and intermittent attacks, the normal temperature, and the normal pulse at intervals of ease. With the above caution you cannot mistake it for other forms of bowel-trouble.

**Tennessee has recently established a State veterinary organization.**

*Treatment.* In no disease so much as in spasmodic colic are the powers of nature and scope of medicine so clearly exemplified; hence the necessity of the practitioner being hasty to reach his patients for fear of arriving too late. Regardless of the different modes of treatment the patient has been subjected to by neighbors —all of whom have a prescription to which they attribute great curative powers, but which generally are not sufficiently injurious to prove fatal, and this stubborn resistance of nature is in itself not always sufficient to effect a favorable termination, I administer two ounces of aloetic mass, followed by one ounce of chloral hydrate dissolved in one pint of water, and use the injection pump freely. This mode of treatment is very effective in this disease.

FLATULENT COLIC is due to a distention of the large intestine with gases, caused by indigestible food, diseased food, cribbing, etc. It is closely allied in many ways to spasmodic colic; in fact, often accompanied by it. The symptoms are usually not so suddenly developed; the abdomen becomes distended and the breathing more labored. The treatment is the same, except that alkalies should be given to neutralize the gases. This disease requires more prompt and persistent treatment than spasmodic colic, and is somewhat more fatal, owing to the interference with the function of some of the internal organs by the abnormal pressure. When the bowels become so distended as to greatly distress the animal do not hesitate to use the trocar and canula in the most distended portion, which usually affords immediate relief. Repeat this until the animal is permanently relieved.

**You should enter the open doors and warmly greet this infant organization.**

